# Effects of Three Semen Extenders, Breeding Season Month and Freezing–Thawing Cycle on Spermatozoa Preservation of Portuguese Merino Sheep

**DOI:** 10.3390/ani11092619

**Published:** 2021-09-07

**Authors:** Margarida Fernandes, Pablo Rodríguez Hernández, João Simões, João Pedro Barbas

**Affiliations:** 1 Department of Veterinary Sciences, Veterinary and Animal Research Centre (CECAV), School of Agricultural and Veterinary Sciences, University of Trás-os-Montes and Alto Douro (UTAD), 5000-801 Vila Real, Portugal; al60050@utad.eu; 2 Department of Animal Production, International Agrifood Campus of Excellence (ceiA3), University of Córdoba, Campus de Rabanales, 14014 Córdoba, Spain; v22rohep@uco.es; 3 Deparment of Biotecnology and Genetic Resources of Instituto Nacional de Investigação Agrária e Veterinária, Quinta da Fonte Boa, 2005-048 Vale de Santarém, Portugal; pedro.barbas@iniav.pt

**Keywords:** ram, frozen semen, semen extender, cryopreservation, sperm viability

## Abstract

**Simple Summary:**

Spermatozoa (SPZ) viability, morphology and the kinetics of fresh and thawing Portuguese Merino Semen were evaluated at different breeding season months using an owner egg yolk-based semen extender (S-EXT) and two commercial lecithin-based semen extenders. The main significant differences between S-EXT were observed for thawed semen. The viability (30% vs. 15% of alive SPZ; *p* < 0.001), total motility (74% vs. 34%; *p* < 0.001), total progressive motility (18% vs. 5%; *p* < 0.001), straight line velocity (37 vs. 22 μm/s; *p* < 0.001) and beat cross frequency (13 vs. 6 Hz; *p* < 0.05) were higher in the owner egg yolk than in egg yolk-free-based S-EXT. Significant interactions between S-EXT, semen processing and/or months of semen collection were observed on several of the 22 evaluated SPZ parameters and should be elucidated in further studies. The egg yolk-based S-EXT was more consistent across the months in cryopreserving SPZ than commercial egg yolk- free-based S-EXT and still seems, to the present day, the most appropriate S-EXT to be used in (Merino) sheep.

**Abstract:**

This study aimed to evaluate and compare the effect of three semen extenders (S-EXT) on 22 spermatozoa (SPZ) parameters (subjective and computer-assisted sperm analysis evaluations), before and after semen cryopreservation throughout different months of the breeding season in the Portuguese Merino breed. According to the multivariable model, the SPZ viability (alive %), kinetics subjective individual motility, total motility, total progressive motility and its subpopulations, and beat cross frequency) were higher in the egg yolk-based S-EXT improved by Estação Zootécnica National (Portugal) than in Ovixcell^®^ or Andromed^®^ extenders. All the differences were only observed in thawed semen, except for total motility and total progressive motility, in which Ovixcell^®^ also showed the poorest results on fresh semen. An interaction effect between S-EXT and semen processing was observed on 72.3% (17/22) of the evaluated parameters, evidencing a variable cryoprotective action between S-EXT. The SPZ viability was poorer in the onset of the breeding season (end of April/early May) than in the previous middle breeding season (November/early December), suggesting the influence of a short anoestrous season on ejaculate quality, even though the volume and SPZ concentration of the ejaculates remained stable throughout the experiment. Additionally, S-EXT x semen processing x month interaction effect on 59.1% (13/22) of the evaluated parameters evidenced the importance of SPZ time collection in a natural environment to cryopreserve ram’s semen. We concluded that, overall, the egg yolk-based S-EXT provided a greater value to the cryopreservation of Merino rams´ semen. Nevertheless, the causes of the interaction effect between S-EXT, semen processing and/or month on several SPZ parameters should be addressed, including SPZ molecular research in new studies, in order to improve egg yolk-based as well as in egg yolk-free-based S-EXT.

## 1. Introduction

Cryopreservation of spermatozoa (SPZ) has several advantages apart from the long-term conservation, such as a cost reduction (e.g., decreasing the number of rams in the flock), easy storage, the prevention of genetic drift and the promotion of genetic resource exchange and transportation all over the world [1,2,3,4,5].

Although cryopreservation has many positive aspects, the cooling process also involves fast structural changes in SPZ, which can promote different cell damages such as the reduction in the membrane fluidity or the alteration of lipid and protein organization [6,7,8]. In view of the above, semen is usually diluted before cryopreservation using freezing semen extenders (S-EXT), which can be formulated using different components such as Tris, fructose or glucose, citric acid, glycerol, antibiotics, and egg yolk (EY) [3,4]. In sheep, different compounds have been used to supplement S-EXT: enzymes, vitamins, amino acids, proteins, some plant extracts and other compounds such as sugars, seminal plasma and fatty acids [9]. The main objective of these substances in most cases is the reduction in the oxidative process [10], regarding the regulation, suppression or prevention of reactive oxygen species formation [11,12], which have been shown to directly influence fertilization SPZ capacity [9].

Although artificial insemination and SPZ cryopreservation constitutes a useful tool employed in breeding programs, this methodology is not widely used among animal species [13]. In sheep, there are two main limitations, namely the difficulty for the SPZ to cross the ewe cervix and reach the uterus for fertilization [14,15]; and the complex anatomy of the cervix which impedes vaginally depositing semen directly into the uterus [16]. The results obtained for the fertility rate of vaginal-cervical insemination with frozen–thawed semen have been considered insufficient for commercial breeding programs [13]. Against that background, several studies have been carried out to overcome this difficulty, optimizing methods to cross the cervical barrier [17,18,19] or improving the sperm [9,18,20]. In this sense, many studies [21,22,23,24,25] have been published evaluating different S-EXT and improving its effectiveness in order to reach an acceptable fertility in commercial flocks. At Estação Zootécnica Nacional (EZN), Instituto Nacional de Investigação Agrária e Veterinária, IP (Portugal), an original Australian S-EXT [1] has been studied and gradually improved during the last three decades. Preliminary results obtained in our laboratory [26] suggest that it could preserve several SPZ parameters evaluated after a freezing–thawing cycle.

The beginning of ovine reproduction, as seasonal breeders, is regulated by an increasing photoperiod [27] which takes place between February and August. Although the reproductive regression is more pronounced in the ewe [28,29], this period is also characterized in rams by a reduction in testicular size, libido, sperm quality and quantity, triggering a period of low productivity [30,31]. The research carried out in this sense has noted that the non-breeding season of local sheep breeds is less accentuated or not present in those areas with low latitude [32]. Thus, this period in countries in southern Europe can be shortened to about three months (February to April) when compared with high latitude regions [33]. This fact allows the use of rams during most of the year for semen collection in reproduction centers in a natural environment or under photoperiod stimulation [34], without using expensive hormonal treatments.

The main objective of this study was to compare the effects of EZN S-EXT with two other commercial S-EXT on SPZ thawing quality, evaluated by 22 semen parameters after a freezing–thawing cycle and according to the breeding month in Portuguese Merino sheep.

## 2. Materials and Methods

### 2.1. Animals and Housing

The experiment was performed at EZN (lat: 39°11′57.3″ N′ log: 8°44′22.5″ O′′) during the 2020 breeding season (November and December) and the onset of the 2021 breeding season (late April and early May). Two fertility-proven Merino rams, aged 7 years, were used and maintained under natural environment conditions. Both rams were grouped in a collective park with the same diet: ad libitum hay and a commercial feed concentrate (1 kg per day). Body condition scores varied between 3.5 and 4 (on a 5-point scale) during the whole experiment. Rams were screened for brucellosis and the flock was classified as brucellosis-free. Both rams were also periodical dewormed and vaccinated against clostridial and pasteurellosis diseases.

### 2.2. Semen Collection

At least one ejaculate was obtained one week before the onset of each period (November 2020 and April 2021) in order to preliminarily assess the ejaculate quality and to remove old semen portions from reproductive tract. Then, a total of 8 sessions of semen collection were performed, once per week: November 18th and 25th, December 3rd and 10th, April 22nd and 29th, and May 6th and 11th.

The ejaculates were collected by artificial vagina, immediately maintained in a water bath at 30 °C, and finally evaluated according to the method described by Evans and Maxwell [1]. Ejaculates with poor quality (individual motility, IM < 55%) and/or reduced volume, less than 0.4 mL, were rejected (*n* = 3) and occurred due to non-identified factors. New semen collection was provided in the same day for those situations. All the three new semen collections reached ≥55% of IM and >0.4 mL of volume.

No differences (*p* > 0.05) of SPZ concentration in ejaculates were observed between rams or between months (overall mean ± SEM: 4.9 ± 0.20 × 10^9^ SPZ/mL). Nevertheless, one of the rams produced more ejaculate volume during the whole period of collection sessions (overall means: 1.12 ± 0.06 mL vs. 0.71 ± 0.09 mL; *p* < 0.01).

### 2.3. Semen Extenders

Three S-EXT were used to dilute the semen. Each ejaculate was divided into three aliquots of equal volume and each one was diluted with a different S-EXT. The composition of each aliquot was the following:

1. EZN S-EXT. Currently used at EZN lab, it was adapted from Evans and Maxwell [1]. The composition was 15% of EY; 6% of glycerol; 21,805 g of Tris; 0.3 g of Glucose; 1194 g of citric acid; 0.05 g of Penicillin and 38 mL of sterile bi-distilled water. The EY was obtained from daily fresh chicken eggs. The EY was manually separated from albumen using a filter paper and a sterile syringe to pierce the chalaza.
2. Andromed^®^ (ANDR) S-EXT (Minitüb, Tiefenbach, Germany). It was an EY-free concentrated extender medium composed by phospholipids, TRIS, citric acid, sugars, antioxidants, buffers, glycerol, purest water and antimicrobials (tylosin, gentamicin, spectinomycin and lincomycin), according to the manufacturer details.
3. Ovixcell^®^ (OVIX) S-EXT (IMV technologies, L’Aingle, France). This S-EXT was soybean lecithin-based [34]. No manufacturer details were available at the current date.

Both commercial S-EXT were stored in the original flask at 5 °C during the whole assay. The EZN S-EXT was prepared on the previous day of each session. At semen collection time, a portion of each S-EXT was maintained on water bath at 30 °C.

### 2.4. Semen Processing

Semen volume was immediately measured with a graduate collection vial. The semen concentration was determined by spectrophotometry (WPA-S106). Pooled semen was divided into three aliquots of equal volume and diluted with each S-EXT in the water bath at 30 °C to obtain a final concentration of 1 × 10⁸ SPZ/mL. The diluted fresh semen was evaluated and packed in mini straws of 0.25 mL in a water bath at 28 °C, and then cooled at 5 °C for 4 h. Afterwards, the straws in the rack were exposed to liquid nitrogen vapors at −120 °C for 20 min. Finally, the straws were plunged into a nitrogen liquid tank at −196 °C.

After one week, the straws were thawed in a water bath at 38 °C for 1 min. The content of the straws was diluted in 1 mL of saline solution and homogenized for 90 s. After two minutes, thawed semen parameters were evaluated for all the semen processing. Evaluation processes were performed by the same person throughout the whole study.

### 2.5. Semen Evaluation

IM (%) was determined subjectively by using a phase-contrast microscope (200× magnification) with a warm slide (37 °C). Sperm viability (alive; %) and normality (normal and abnormal; %), head defects (%), tail defects (%) and middle piece defects (MP; %) were evaluated by a smear stained of nigrosine-eosin (magnification of 1000×; Olympus BX40 microscopic^®^, Tokyo, Japan).

Computer-assisted sperm analysis (CASA) system (ISASv1^®^, Valencia, Spain) was used to determine the SPZ motility parameters for rams. A 10 µL drop of diluted semen was placed and covered with a coverslip on a warm slide (37 °C). For each sample, 5 image repetitions were made. The percentage of total motile sperm cells (TM; %), total progressive motility (TPM; %), total static (TS; %), subpopulations of TPM (slow, medium and rapid; %), curvilinear velocity (VCL; μm/s), straight-line velocity (VSL; μm/s), average-path velocity (VAP; μm/s), linearity (LIN ¼ [VSL/VCL] × 100; %), amplitude of lateral head displacement (ALH; μm), straightness (STR[VSL/VAP] × 100; %), wobble (WOB [VAP/VCL] × 100; %) and beat cross frequency (BCF; Hz) were evaluated.

### 2.6. Statistical Analysis

The Shapiro–Wilk W test was used to evaluate the data distribution and an arcsine square root transformation was carried out to approach or reach normality. A multivariable univariate model for repeated measures was built considering each SPZ parameter. Restricted maximum likelihood (REML) method was used to fit the linear mixed models. The S-EXT (EZN, ANDRO and OVIX), months (November, December, April and May) and semen processing (fresh and thawed semen) variables were considered as fixed effects and animal variable as a random effect. First- and second-way interactions of the fixed variables were also included in the models. Tukey’s test was used to evaluate the differences between pairs.

All data were analyzed using the software JMP^®^ 14 for Windows (SAS Institute, Cary, NC, USA). Results were presented as Least square mean ± (square root transformed) standard error mean for a significance level of 0.05.

## 3. Results

Overall, all SPZ parameters were affected by semen processing: 40.1% (9/22) by S-EXT and 40.1% (9/22) by month of semen collection (Table 1). As expected, the majority of poor values was observed in the thawed semen. Several one-way and two-way interactions were observed between month, S-EXT and/or semen processing.

The SPZ viability (alive %) was higher (*p* < 0.01) using EZN or ANDR S-EXT than OVIX (Table S1). Higher values of IM (*p* < 0.01), TM (*p* < 0.05), TPM (*p* < 0.01), medium and rapid TPM subpopulations (*p* < 0.01; Table S2), VSL (*p* < 0.05; Table S3), were observed in EZN than OVIX or ANDR S-EXT in thawed semen.

**Figure 1:**
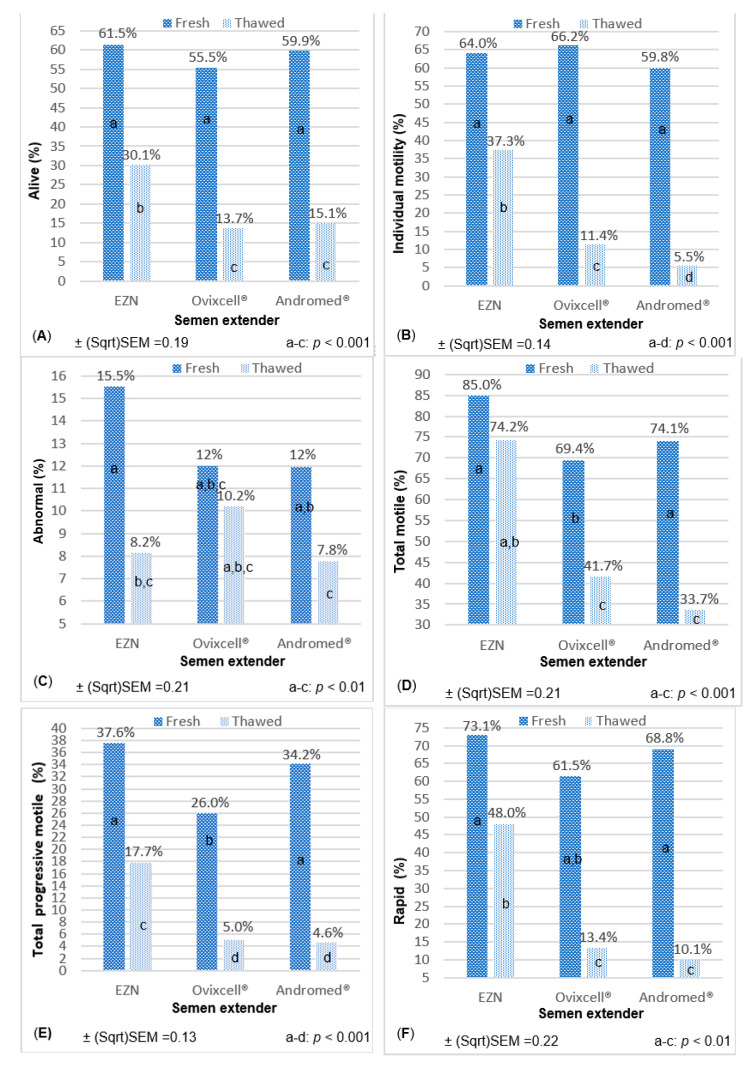

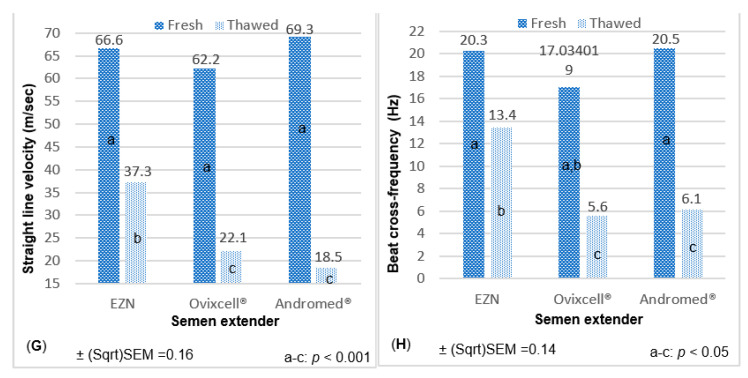
Semen extender X semen processing interaction effects on selected spermatozoa parameters. (**A**) Similar values of spermatozoa (SPZ) viability were observed on fresh semen between different semen extenders (S-EXT), but a less pronounced decrease of the mean percentage of alive SPZ occurred after thawing in EZN S-EXT than in Ovixcell® (OVIX S-EXT) or Andromed® (ANDR S-EXT) extenders, (**B**) the mean percentage of individual (subjective) motility SPZ in fresh semen was independent of S-EXT, but differently affected by freezing-thawing cycle according to the S-EXT, (**C**) contrarily to EZN S-EXT and ANDR S-EXT, no statistical differences of SPZ morphology (mean percentage of abnormal SPZ) was observed between fresh and thawed semen using the OVIX S-EXT. Nevertheless, the mean percentage of abnormal SPZ in fresh semen using OVIX S-EXT was similar to the mean value observed in thawed semen using EZN S-EXT, (**D**) the mean percentage of total motile SPZ (computer-assisted sperm analysis method) in fresh semen was lower using the OVIX S-EXT than EZN S-EXT or ANDR S-EXT. No statistical differences of total motile SPZ were observed between fresh and thawed semen using the EZN S-EXT. Moreover, the mean value of total motile SPZ of the thawed semen using the EZN S-EXT was also similar to the mean values of fresh semen using the other two S-EXT, (**E**) the mean percentage of total progressive motile SPZ was lower in fresh semen using the OVIX S-EXT than using the other two S-EXT. After thawing, the highest value was observed using the EZN S-EXT, (**F**) the mean percentage of rapid SPZ subpopulation of the fresh semen was independent of S-EXT. After thawing, this value is higher using EZN S-EXT than the other two S-EXT. No statistical difference of rapid SPZ subpopulation was observed between thawed semen using EZN S-EXT and fresh semen using OVIX S-EXT, (**G**) Similar mean values of straight-line velocity (μm/s) SPZ were observed between the different S-EXT in fresh semen, but a less pronounced decrease occurred after thawing in EZN S-EXT than in OVIX S-EXT or ANDR S-EXT, (**H**) the interaction effect of semen extender X semen processing on beat cross frequency (Hz) was similar to the observed on the rapid SPZ subpopulation.

The S-EXT x semen processing interaction was the most frequent interaction (72.3%; 17/22) observed in the study (see Table 1). Overall, the EZN S-EXT allowed a better freezing SPZ preservation as reported in Figure 1.

Overall, the month influenced (*p* < 0.05) IM (lowest values in May), alive (highest and lowest values in November and April, respectively), death (highest and lowest values in November and May, respectively), normal (lowest values in December), abnormal (lowest values in May), head (highest and lowest values in April and May, respectively), tail (highest and lowest values in November and May, respectively), medium (highest and lowest values in November and May) (see Table S1) and ALH (highest and lowest values in November and May, respectively) parameters (see Table S3).

## 4. Discussion

About less than 30% of ram SPZ remains biologically undamaged after thawing [35]. Membrane integrity constitutes one of the main features which make the functionality of the preservation of spermatozoa difficult. Among sperm cell components, three membranes can be distinguished: plasmatic and mitochondrial membranes, which are involved not only in sperm viability and motility, but also in the process of capacitation; and the acrosome membrane, responsible for penetrating the oocyte [36]. EY has been a common constituent of semen extenders, protecting the spermatozoa against cold shock and seminal plasma proteins, and conferring protection during freezing and thawing. The low density lipoproteins of EY act at cell membrane level [35,37] at least in bulls and probably in rams, they reduce the adverse activity of plasma seminal [38], replacing damaged phospholipids of the membrane [39]. The low-density lipoprotein is the EY element that provides protection for plasma membrane integrity in cryopreservation [40].

In the last decade, replacing it with soybean lecithin constituent has been a viable alternative in order to avoid external contaminations and SPZ agglutination [41,42,43,44,45], but the protection mechanism of soybean for sperm cells is not yet fully known. In this sense, while some studies indicate that soybean lecithin creates a protective film around the spermatozoa [46], others suggest that soybean lecithin phospholipids replace those altered in sperm cell membrane [47]. Nevertheless, the cryoprotection differences observed in this study between EY and soybean lecithin-based S-EXT were not always evident [48,49]. Lyophilized EY-based S-EXT has been evaluated to keep its utility for long term use in reproduction centers [21] or even when its composition is supplemented with L-carnitine [50].

In the present study, we observed better results for EZN than for commercial S-EXT (alive %, IM, TM %, TPM %, TS %, rapid subpopulation, VSL and BCF) after semen thawing. ANDR is a cattle S-EXT-based soy lecithin whose application to ram semen preservation have been successfully evaluated in the last year after chilled semen processing at 15 °C [51] or at 5 °C [52]), allowing a 65-day pregnancy rate higher than 70% after intracervical insemination (95% confidence interval 64.7 to 76.9% [52]). Additionally, fertility (pregnancy rate = 51% after laparoscopic intrauterine insemination [52]) and in vitro insemination studies [49,53,54] have been published regarding thawed semen. [49] We observed similar SPZ viability (39.7%), TM (28.8%) and TPM (44.8%) between ANDR and EY-based S-EXT (*p* > 0.05). In our study, we observed that the percentage of alive SPZ (30.1%) using EZN S-EXT was more than double both soybean lecithin-based S-EXT (OVIX—13.7%; ANDR—15.1%), and was similar to the results reported by Savvulidi et al. [54]. The same high values were observed regarding TM (74.2%) and TPM (17.7%) values using EZN S-EXT in the present study. Additionally, VSL was higher in EZN (>35 mm/s; *p* < 0.001) than the other S-EXT. ANDR^®^ demonstrated17.0 mm/s, which was less than OVIX (about 22 mm/s), and similar to the results of the study performed by [53] (VSL = 13.2 mm/s).

According to Del Olmo et al. [55], the kinetic parameter evaluation by CASA is one of the main methods for assessing the fertility potential of a ram’s ejaculate. Using an owner freezing extender in white Manchega sheep breed, they observed the following results in thawed semen (mean±SEM): TM = 83.8 ± 3.8%, TPM = 24.8 ± 4.2%, VAP = 0.1 ± 3.9 um/s, VCL = 89.5 ± 2.5 um/s, VSL = 49.7 ± 6.5, LIN = 47.0 ± 3.9%, ALH = 2.9 ± 0.2 um, and BCF = 4.9 ± 0.2 Hz. Only results obtained with EZN S-EXT were close to these TM, TPM and VSL values. In our study, VCL, VAP, LIN and ALH were not affected by S-EXT, but their S-EXT x semen processing interactions were significant, showing a higher activity of EZN S-EXT. Additionally, a higher BCF (>12 Hz; *p* < 0.01) was observed using EZN than using lecithin-based S-EXT, which is in agreement with the study by Del Olmo et al. [55]. Other studies explored the associations of the CASA motility parameters with functional tests such as the migration efficiency in cervical mucus or fertility trials. VAP, VSL, VCL and ALH are positively associated with fertility after intra-uterine insemination in ram semen [56]. Both VAP and VCL were shown to be good indicators of the capacity of spermatozoa to migrate in sheep cervical mucus [57].

Overall, all the three S-EXT showed similar values of SPZ parameters in fresh semen except for TM (Figure 1D) and TPM (Figure 1E), in which a decrease of about 15% was observed for OVIX EXT (*p* < 0.001). Our findings contrasted with the recent study of Khatun et al. [58], in which a higher SPZ motility was observed using OVIX compared to the use of an EY-based S-EXT in both the pre-freeze (fresh semen) and thawing stages. Even if our study can suggest that the lower TM and TPM after thawing can be partially attributed to the low values using the OVIX S-EXT, similar values in thawed semen were observed between OVIX and ANDR S-EXT. This fact suggests that other factors related to the S-EXT take place during the freezing–thawing cycle. For example, season can affect the seminal plasma proteins bounded to SPZ, which can change SPZ motility in thawed semen [59]. Kulíková et al. [60] reported that OVIX S-EXT supplemented with trehalose enhanced SPZ kinetic. To a reader it would be easier to understand the effects of OVIX S-EXT if we knew its composition. Therefore, the full constitution should be published to evaluate its components and percentages.

It is already known that rams’ reproductive seasonality can be marked by a decrease in fresh semen parameters in the anestrous season in higher latitudes [61]. A decrease in semen quality (parameters) among breeds at temperate and moderate latitudes has been reported [62,63]. In our study, no significant differences in fresh semen were detected between the months, which can be justified by the superficial anestrous from the Merino breed and also by the use of two experienced rams with good semen quality and in vivo fertility. Our multivariable model has highlighted not only the differences between S-EXT and semen processing but also the timeline profile at full breeding season (November and December) and the onset of the next early breeding season (April and May). The several significant one-way and two-way interactions between month, S- EXT and/or semen processing (see Table 1) also provided a global overview about the pattern of the semen parameter results. Continuous breeding of local breeds, even with differences in ejaculate quality between seasons were previously observed in low latitudes [64]. However, a little influence of seasonality in semen quality can partially justify the monthly differences (and its interactions with S-EXT and/or semen processing). In thawed semen, significant differences between months were observed concerning IM, alive, abnormal, head and tail parameters (see Table S1). The highest differences in alive, head and tail parameters in thawed semen were observed in April only for EZN S-EXT. Nevertheless, in April, the alive parameter (SPZ viability) was significantly higher for EZN (20%) than the other OVIX (13%) and ANDR (12%) S-EXT ensuring it profitability.

## 5. Conclusions

Based on the results of our study, we conclude that, based on IM and CASA parameters, thawed semen from Merino rams previously diluted with EZN S-EXT extender had higher quality compared with the commercial S-EXT. Additionally, EZN S-EXT was more consistent for the cryopreservation of SPZ throughout the different months of semen collection.

Further studies are needed to improve and optimize cryopreservation protocols with extenders without products of animal origin. In addition, the causes of interaction between S-EXT, semen processing and months with seminal parameters should be encouraged in future research.

The non-significant differences between the several SPZ parameters in fresh semen across the studied months, which were associated with a constant volume of the ejaculate, allow this local Merino breed to be consistently used for reproduction purposes at our latitudes.

## Figures and Tables

**Table 1:** Effect of fixed variables (month, semen extender and semen processing) and their one-way and two-way interactions.

Parameters	Fixed Variables			Interactions			
	M	S-EXT	SP	M × S-EXT	M × SP	S-EXT × SP	M × S-EXT × SP
Alive	***	*	***	***	***	***	**
Death	***	NS	***	***	NS	NS	NS
IM	***	**	***	***	***	***	**
Normal	***	NS	*	***	**	NS	***
Abnormal	***	NS	***	***	NS	***	NS
Head	***	NS	***	*	***	NS	NS
MP	NS	NS	***	***	NS	NS	NS
Tail	***	NS	**	***	***	***	***
TM	NS	*	***	NS	NS	***	NS
TS	NS	**	***	*	NS	***	*
TPM	NS	**	***	NS	NS	***	NS
Slow	NS	NS	***	NS	NS	***	***
Medium	**	**	***	NS	***	NS	*
Rapid	NS	**	***	NS	NS	***	*
VCL	NS	NS	***	NS	***	***	*
VSL	NS	*	***	NS	*	***	NS
VAP	NS	NS	***	NS	**	***	NS
LIN	NS	NS	***	NS	NS	**	***
ALH	***	NS	***	NS	**	***	NS
STR	NS	NS	***	*	NS	*	***
WOB	NS	NS	*	**	NS	**	***
BCF	NS	*	***	NS	NS	***	**

M, month; S-EXT, semen extender; SP, semen processing; IM, individual motility; MP, middle piece; TM, total motility; TPM, total progressive motility; TS, total static; VCL, curvilinear velocity; VSL, straight-line velocity; VAP, average-path velocity; ALH, amplitude of lateral head displacement; LIN, linearity; STR, straightness; WOB, wobble; BCF, beat cross frequency. *—*p* < 0.005; **—*p* < 0.01, ***—*p* < 0.001; NS, not significant (*p* > 0.05).

## Data Availability

The data that support the findings of this study are available on request from the corresponding author (J.S.).

## Supplementary Materials

The following are available online at https://www.mdpi.com/article/10.3390/ani11092619/s1, Table S1: Effects of semen extender, month and thawing according to their interactions on the percentage of individual motility (IM), viability (alive) and morphology of spermatozoa, Table S2: Effects of semen extender, month and thawing according to their interactions on the percentage of total motility (TM), total motility progressive (TPM) and its subpopulations, and total static (TS) of spermatozoa (CASA), Table S3: Effects of semen extender, month and thawing according to their interactions on the additional spermatozoa kinetic parameters (CASA).
