# Supplementary material for: Effects of Three Semen Extenders, Breeding Season Month and Freezing–Thawing Cycle on Spermatozoa Preservation of Portuguese Merino Sheep
Source: Animals (Basel). 2021 Sep 7;11(9):2619. doi: 10.3390/ani11092619 (PMC8467096; doi:10.3390/ani11092619)
Supplement: Supplementary file 1 [file animals-11-02619-s001.zip › animals-1343653-supplementary.pdf]

animals

MDPI

Table S1. Effects of semen extender, month and thawing according to their interactions on the percentage of individual motility (IM), viability (Alive) and morphology of spermatozoa.

S-EXT	Month	SP	Spermatozoa (%)							
			**Alive**	**Death**	**IM**	**Normal**	**Abnormal**	**Head**	**MP**	**Tail**
EZN	Nov	Fresh	61.2^{a}	37.9^{g,h}	66.2^{a}	78.5^{a}	19.5^{a}	1.1^{b,c,d,e,f,g,h}	6.0^{a,b,c,d,e,f,h,i,j}	9.3^{a,b}
		Thawed	33.1^{c,d}	66.3^{b,c,d}	35.1^{b}	89.5^{a,b}	10.0^{b,c,d,e,f}	0.7^{b,c,d,e,f,g,h}	2.1^{f,g,h,i,j,k,l}	6.9^{a,b,c,d,f,g,i}
	Dec.	Fresh	67.4^{a}	31.6^{h}	63.7^{a}	83.7^{a,b}	14.9^{a,b,c,d}	0.9^{b,c,d,e,f,g,h}	7.7^{a,b,c,d,e}	4.9^{c,d,e,f,g,h,i,j,k}
		Thawed	33.4^{c,d}	73.0^{a,b,c}	37.7^{b}	60.3^{c}	8.2^{d,e,f}	0.9^{b,c,d,e,f,g,h}	0.5^{l}	9.5^{a,b}
	April	Fresh	53.9^{a}	45.8^{e,g}	62.5^{a}	82.9^{a,b}	16.7^{a,b,c}	1.1^{b,c,d,e,f,g,h}	7.5^{a,b,c,d,e}	7.7^{a,b,c,f,g}
		Thawed	19.6^{e,f}	79.9^{a,b,c}	38.0^{b}	87.9^{a,b}	8.8^{d,e,f}	3.6^{a}	1.4^{g,k,l}	3.4^{e,h,j,k,l}
	May	Fresh	63.9^{a}	35.2^{g,h}	63.7^{a}	87.4^{a,b}	11.7^{a,b,c,d,e,f}	0.5^{e,f,g,h}	6.2 ^{a,b,c,d,e,h,i}	4.3^{d,e,h,i,j,k,l}
		Thawed	36.0^{b,c,d}	63.6^{b,c,d,f}	39.5^{b}	93.7^{b}	6.0^{e,f}	0.9^{b,c,d,e,f,g,h}	0.9^{k,l}	3.0^{h,j,kl}
ANDR	Nov	Fresh	58.0^{a}	36.9^{g,h}	70.0^{a}	91.5^{a,b}	8.5^{b,c,d,e,f}	1.4^{a,b,c,d,e}	3.0^{b,d,e,f,g,h,i,j,k,l}	3.9^{f,h,I,j,k,l}
		Thawed	27.7^{d,e}	70.2^{b,c}	13.6^{c,d}	90.7^{a,b}	9.2^{a,b,c,d,e,f}	0.9^{c,e,f,g,h}	1.5^{h,i,j,k,l}	6.4^{a,b,c,d,f,g,h,j}
	Dec	Fresh	59.9^{a}	39.2^{g,h}	65.0^{a}	83.8^{a,b}	12.9^{a,b,c,d,e,f}	0.6^{e,f,g,h}	8.6^{a,c}	2.7^{i,kl}
		Thawed	12.9^{f,g}	86.4^{a,b,c}	11.9^{c,d}	89.0^{a,b}	8.0^{b,c,d,e,f}	1.2^{a,b,c,d,e,g}	2.9^{b,d,e,f,g,h,i,j,k,l}	6.5^{a,b,c,d,f,g,h,j}
	April	Fresh	49.5^{a,b,c}	48.3^{d,e,f,g,h}	63.7^{a}	85.1^{a,b}	13.7^{a,b,c,d,e}	1.0^{b,c,d,e,g}	6.4^{a,b,c,d,e,f,g}	4.7^{b,f,g,h,i,j,k}
		Thawed	12.2^{f,g}	87.5^{a,b,c}	18.2^{c}	87.2^{a,b}	12.4^{a,b,c,d,e,f}	2.8^{a,b,d}	1.5^{h,i,j,k,l}	6.9^{a,b,c,d,e,f,g,h}
	May	Fresh	54.8^{a,b}	42.3^{g,h}	66.1^{a}	85.6^{a,b}	13.3^{a,b,c,d,e,f}	0.1^{f,h}	6.5^{a,b,c,d,f,g}	6.6^{a,b,c,d,f,g,h,j}
		Thawed	5.9^{g}	92.3^{a}	4.4^{e,f}	88.2^{a,b}	11.5^{a,b,c,d,e,f}	0.4^{e,f,g,h}	1.9^{e,h,i,j,k,l}	8.4^{a,c,d,e}
OVIX	Nov.	Fresh	63.2^{a}	36.6^{g,h}	65.0^{a}	82.5^{a,b}	16.3^{a,b,d}	1.2^{a,b,c,d,e}	9.2^{a,b}	4.4^{a,b,c,d,f,g,h,i,j}
		Thawed	21.7^{d,e,f}	77.3^{a,b,c}	3.4^{f}	89.3^{a,b}	10.8^{a,b,c,d,e,f}	0.7^{d,e,f,g,h}	2.7^{c,d,e,f,g,h,i,j,k,l}	6.1^{a,b,c,d,e,f,h,i}
	Dec.	Fresh	56.5^{a}	43.0^{f,g,h}	56.2^{a}	86.1^{a,b}	11.2^{a,b,c,d,e}	1.7^{a,b,c,d,e}	6.2^{a,b,c,d,e,f,g,h}	3.0^{g,j,kl}
		Thawed	13.2^{f,g}	82.5^{a,b}	8.2^{d,e}	91.7^{a,b}	8.0^{c,e,f}	1.5^{a,b,c,d,e}	1.9^{d,e,h,i,j,k,l}	4.1^{a,b,c,d,e,f,h,i,j}
	April	Fresh	55.8^{a}	43.7^{f,g,h}	55.9^{a}	87.1^{a,b}	12.1^{a,b,c,d,e}	1.7^{a,b,c,d,e}	5.5^{a,b,c,d,e,f,g,h,j}	4.2^{a,b,c,d,e,f,h,i,j}
		Thawed	12.7^{f,g}	87.3^{a,b}	13.7^{c,d}	91.4^{a,b}	7.8^{c,e,f}	2.9^{a,b,c}	2.3^{d,e,f,g,h,i,j,k,l}	1.7^{kl}
	May	Fresh	64.2^{a}	35.7^{g,h}	62.5^{a}	89.6^{a,b}	8.8^{b,c,d,e,f}	0.1^{g,h}	5.1^{a,b,c,d,e,f,g,h,j,k}	1.2^{l}
		Thawed	13.5^{f,g}	64.6^{c,d,e}	1.0^{f}	95.0^{a,b}	5.0^{f}	0.7^{d,e,f,g,h}	1.2^{k,l}	2.2^{j,kl}
±(Sqrt)SEM			±0.27	±0.28	±0.17	±0.20	±0.28	±0.17	±0.27	±0.21

$^{a-k}$ different superscript letters in the same column: $p < 0.05$. S-EXT.: semen extender; SE- semen processing; EZN- dilutor used at Estação Zooténica Nacional; OVIX- Ovixcell®; ANDR- Andromed®; MP – Middle piece. Highest and lowest averaged least square means in bold.

Table S2. Effects of semen extender, month and thawing according to their interactions on the percentage of total motility (TM), total motility progressive (TPM) and its subpopulations, and total static (TS) of spermatozoa (CASA).

EXT	Month	SP	Spermatozoa (%)					
			**TM**	**TS**	**TMP**	**Slow**	**Medium**	**Rapid**
EZN	Nov	Fresh	82.4^{a}	$12.8^{e,f,g}$	$36.0^{a,b,c}$	$4.9^{c,d,e,f,g}$	$4.7^{d,e,f,g,h,i}$	$67.5^{a,b,c}$
		Thawed	83.5^{a}	$11.9^{e,f,g}$	$16.1^{e,f}$	$3.1^{d,e,f,g,h}$	$11.6^{a,b,c,d}$	$62.0^{a,b,c,d}$
	Dec	Fresh	88.9^{a}	8.7^{g}	$35.2^{a,b,c}$	$2.7^{e,f,g,h}$	$5.3^{c,d,e,f,g,h,i}$	76.8^{a}
		Thawed	75.2^{a}	$20.5^{e,f,g}$	$14.8^{f,g}$	$8.0^{a,b,c,d,e}$	16.7^{a}	$45.1^{c,d}$
	April	Fresh	86.7^{a}	$9.5^{f,g}$	40.3^{a}	$4.1^{d,e,f,g,h}$	$6.4^{b,c,d,e,f,g,h}$	$73.7^{a,b}$
		Thawed	$64.3^{a,b,c}$	$27.8^{b,c,d,e}$	$18.2^{d,e,f}$	$9.4^{a,b,c,d,e}$	$7.4^{b,c,d,e,f,g}$	$39.4^{d,e}$
	May	Fresh	82.2^{a}	$14.1^{e,f,g}$	39.2^{a}	$1.5^{f,g,h}$	$3.1^{f,g,h,i}$	74.4^{a}
		Thawed	74.7^{a}	$21.9^{d,e,f,g}$	$22.3^{c,d,e,f}$	$10.1^{a,b,c,d,e}$	$12.2^{a,b,c}$	$46.7^{b,c,d}$
ANDR	Nov	Fresh	$64.7^{a,b}$	$27.1^{b,c,d,e}$	$22.9^{c,d,e,f}$	0.3^{h}	1.7^{i}	$60.5^{a,b,c,d}$
		Thawed	$41.3^{c,d,e}$	54.9^{a}	$6.6^{g,h}$	$13.3^{a,b,c}$	$13.5^{a,b}$	12.8^{f}
	Dec	Fresh	69.6^{a}	$24.0^{c,d,e,f,g}$	$24.6^{b,c,d,e,f}$	$0.9^{f,g,h}$	$3.8^{e,f,g,h,i}$	$63.2^{a,b,c,d}$
		Thawed	$45.3^{b,c,d}$	$49.7^{a,b}$	5.1^{h}	$14.9^{a,b}$	$11.2^{a,b,c,d}$	13.3^{f}
	April	Fresh	72.3^{a}	$24.2^{c,d,e,f,g}$	$28.0^{a,b,c,d,e}$	$4.3^{d,e,f,g,h}$	$6.3^{b,c,d,e,f,g,h,i}$	$58.8^{a,b,c,d}$
		Thawed	$39.5^{d,e}$	55.4^{a}	5.5^{h}	$9.1^{a,b,c,d,e}$	$7.4^{b,c,d,e,f,g}$	$18.9^{e,f}$
	May	Fresh	71.0^{a}	$24.1^{c,d,e,f,g}$	$29.0^{a,b,c,d,e}$	$1.4^{f,g,h}$	$2.4^{g,h,i}$	$63.7^{a,b,c,d}$
		Thawed	$40.8^{c,d,e}$	$47.5^{a,b,c,d}$	3.2^{h}	$14.4^{a,b,c}$	$6.1^{b,c,d,e,f,g,h,i}$	9.6^{f}
OVIX	Nov	Fresh	82.5^{a}	$10.8^{e,f,g}$	$31.9^{a,b,c}$	$0.6^{g,h}$	$2.0^{h,i}$	79.8^{a}
		Thawed	$38.4^{d,e}$	$47.5^{a,b,c}$	5.4^{h}	$14.6^{a,b}$	$9.7^{a,b,c,d,e}$	12.4^{f}
	Dec	Fresh	$70.1^{a,b}$	$26.4^{b,c,d,e,f}$	$29.5^{a,b,c,d}$	$1.5^{f,g,h}$	$3.8^{e,f,g,h,i}$	$62.8^{a,b,c,d}$
		Thawed	$38.3^{d,e}$	59.2^{a}	4.6^{h}	17.4^{a}	$9.6^{a,b,c,d,e}$	8.6^{f}
	April	Fresh	72.0^{a}	$23.6^{d,e,f,g}$	$33.7^{a,b,c}$	$0.9^{f,g,h}$	$3.6^{e,f,g,h,i}$	$64.4^{a,b,c,d}$
		Thawed	$35.5^{d,e}$	60.7^{a}	4.6^{h}	$10.8^{a,b,c,d}$	$8.4^{a,b,c,d,e,f}$	12.4^{f}
	May	Fresh	75.9^{a}	$16.4^{e,f,g}$	42.3^{a}	$0.9^{f,g,h}$	$2.7^{f,g,h,i}$	$68.9^{a,b,c}$
		Thawed	23.7^{e}	65.5^{a}	3.8^{h}	$5.6^{b,c,d,e,f}$	$5.5^{c,d,e,f,g,h,i}$	7.4^{f}
±(Sqrt)SEM			±0.33	±0.41	±0.19	±0.31	±0.23	±0.38

$^{a\text{-}k}$ different superscript letters in the same column: $p < 0.05$. S-EXT: semen extender; SE- semen processing; EZN- dilutor used at Estação zooténica Nacional; OVIX- Ovixcell®; ANDR- Andromed®; Highest and lowest averaged least square means in bold.

Table S3. Effects of semen extender, month and thawing according to their interactions on the additional spermatozoa kinetic parameters (CASA).

EXT	Month	SP	Spermatozoa							
			**VCL**	**VSL**	**VAP**	**ALH**	**LIN**	**STR**	**WOB**	**BCF**
EZN	Nov	Fresh	$173.6^{a,b}$	$58.2^{a,b}$	$81.0^{a,b}$	$3.6^{a,b,c,d,e,f}$	$36.0^{a,b}$	$76.9^{a,b}$	$46.5^{a,b,c}$	20.3^{a}
		Thawed	$142.5^{b,c}$	37.5^{c}	$64.2^{b,c,d}$	$3.1^{a,b,c,d,e,f,g}$	$26.4^{b,c,d}$	$58.6^{d,e,f}$	$45.3^{a,b,c}$	$11.4^{b,c,d,e,f}$
	Dec	Fresh	$179.0^{a,b}$	66.5^{a}	90.0^{a}	$3.6^{a,b,c,d,e,f}$	$34.3^{a,b,c}$	$73.6^{a,b,c,d,e}$	$46.4^{a,b,c}$	21.9^{a}
		Thawed	$107.6^{c,d,e,f}$	$32.2^{c,d}$	$53.3^{d,e,f}$	$2.6^{a,b,c,d,e,f,g,h}$	$29.3^{a,b,c,d}$	$59.4^{c,d,e,f}$	$45.8^{a,b,c}$	$10.9^{c,d,e,f}$
	April	Fresh	$175.7^{a,b}$	66.3^{a}	$86.2^{a,b}$	$3.4^{a,b,c,d,e,f}$	37.6^{a}	$76.4^{a,b,d}$	$48.9^{a,b}$	$19.1^{a,b,c}$
		Thawed	$116.2^{c,d}$	38.9^{c}	$56.8^{c,d,e}$	$2.7^{a,b,c,d,e,f,g,h}$	$32.9^{a,b,c,d}$	$67.2^{a,b,c,d,e,f}$	$48.5^{a,b}$	$15.2^{a,b,c,d}$
	May	Fresh	211.3^{a}	75.8^{a}	99.3^{a}	$3.8^{a,b,c,d,e,f}$	$35.9^{a,b}$	$76.8^{a,b}$	$46.7^{a,b,c}$	$19.9^{a,b}$
		Thawed	$114.3^{c,d,e}$	$40.9^{b,c}$	$58.2^{c,d}$	$2.6^{a,b,c,d,e,f,g,h}$	$35.5^{a,b}$	$69.6^{a,b,c,d,e,f}$	$51.0^{a,b}$	$16.8^{a,b,c}$
ANDR	Nov	Fresh	$213.4^{a,b}$	62.3^{a}	$89.4^{a,b}$	4.2^{a}	$29.7^{a,b,c,d}$	$69.9^{a,b,c,d,e}$	$42.4^{b,c}$	$14.1^{a,b,c,d,e}$
		Thawed	$70.5^{d,e,f,g}$	$21.2^{d,e}$	$34.7^{f,g,h}$	$2.2^{d,e,f,g,h}$	$30.1^{a,b,c,d}$	$61.2^{a,b,c,d,e,f}$	$49.1^{a,b}$	$6.1^{f,g}$
	Dec	Fresh	$194.2^{a,b}$	60.8^{a}	$86.4^{a,b}$	4.1^{a}	$30.3^{a,b,c,d}$	$70.3^{a,b,c,d,e}$	$43.0^{a,b,c}$	$17.4^{a,b,c}$
		Thawed	$70.4^{d,e,f,g}$	$20.8^{d,e}$	$34.9^{f,g,h}$	$2.4^{b,c,d,e,f,g,h}$	$30.1^{a,b,c,d}$	$59.3^{b,c,d,e,f}$	$50.6^{a,b}$	$7.5^{d,e,f,g}$
	April	Fresh	$185.5^{a,b}$	60.9^{a}	$83.4^{a,b,c}$	$3.5^{a,b,c,d,e}$	$32.9^{a,b,c,d}$	$73.2^{a,b,c,d,e}$	$44.7^{a,b,c}$	$19.1^{a,b,c}$
		Thawed	$94.2^{c,d,e,f}$	$25.0^{c,d,e}$	$56.2^{d,e,f,g}$	$1.9^{f,g,h}$	$26.5^{b,c,d}$	$53.4^{f,g}$	$49.8^{a,b}$	$5.7^{f,g}$
	May	Fresh	$198.3^{a,b}$	64.9^{a}	$90.1^{a,b}$	$3.9^{a,b,c}$	$32.1^{a,b,c,d}$	$71.5^{a,b,c,d,e}$	$45.0^{a,b,c}$	$17.8^{a,b,c}$
		Thawed	$65.8^{d,e,f,g}$	$21.6^{d,e}$	$34.8^{f,g,h}$	$0.8^{h,i}$	$34.6^{a,b,c}$	$62.2^{a,b,c,d,e,f}$	55.1^{a}	3.3^{g}
OVIX	Nov	Fresh	214.2^{a}	66.7^{a}	96.1^{a}	$3.7^{a,b,d,f}$	$31.0^{a,b,c,d}$	$69.8^{a,b,c,d,e,f}$	$44.2^{a,b,c}$	$20.6^{a,b}$
		Thawed	$64.2^{e,f,g}$	$20.2^{d,e}$	$33.2^{f,g,h}$	$1.9^{e,g,h,i}$	$32.0^{a,b,c,d}$	$60.6^{d,e,f}$	$52.3^{a,b}$	$6.6^{f,g}$
	Dec	Fresh	$192.7^{a,b}$	66.8^{a}	$89.7^{a,b}$	$3.4^{a,b,c,d,f}$	$34.2^{a,b,c}$	$75.0^{a,b,c,d}$	$46.2^{a,b}$	21.5^{a}
		Thawed	$60.2^{f,g}$	$18.7^{d,e}$	$31.0^{g,h}$	$2.1^{c,e,g,h,i}$	$31.8^{a,b,c,d}$	$60.7^{b,c,d,e,f}$	$52.1^{a,b}$	$7.1^{e,f,g}$
	April	Fresh	$182.2^{a,b}$	66.8^{a}	$86.9^{a,b}$	$3.1^{a,b,c,d,e,f}$	$36.0^{a,b}$	$75.7^{a,b,c,d}$	$47.4^{a,b}$	$20.6^{a,b}$
		Thawed	$79.8^{d,e,f}$	$19.6^{d,e}$	$35.0^{e,f,g,h}$	$1.5^{g,h,i}$	$25.2^{c,d}$	$56.0^{e,f,g}$	$44.5^{a,b,c}$	$7.0^{e,f,g}$
	May	Fresh	$196.5^{a,b}$	77.0^{a}	96.1^{a}	$3.3^{a,b,c,d,f}$	38.4^{a}	79.1^{a}	$48.3^{a,b}$	$19.4^{a,b,c}$
		Thawed	44.0^{g}	15.6^{e}	23.4^{h}	$1.1^{h,i}$	23.4^{d}	42.2^{g}	39.9^{c}	4.2^{g}
±(Sqrt)SEM			±0.52	±0.27	±0.31	±0.12	±0.18	±0.21	±0.19	±0.23

$^{a\text{-}k}$ different superscript letters in the same column: $p < 0.05$. S-EXT: semen extender; SE- semen processing; EZN- Estação Zooténica Nacional; OVIX- Ovixcell®; ANDR- Andromed®; VCL- Curvilinear velocity (μm/sec); VSL-Straight-line velocity (μm/sec); VAP- Average-path velocity (μm/sec); ALH- Amplitude of lateral head displacement (μm); LIN- Linearity (%); STR- Straightness (%); WOB- Wobble (%); BCF- Beat cross frequency (Hz). Highest and lowest averaged least square means in bold.
